# Ultrasonographic Evaluation of Superior Cluneal Nerve Entrapment: Methodological Considerations. Comment on Iudicelli et al. The Role of Musculoskeletal Ultrasound in Detecting Superior Cluneal Nerve Entrapment: Biomechanical Insights in Chronic Low Back Pain—A Pilot Study. *Diagnostics* 2026, *16*, 469

**DOI:** 10.3390/diagnostics16152471

**Published:** 2026-08-05

**Authors:** Busra Sezer Kiral

**Affiliations:** Physical Therapy and Rehabilitation, Istanbul Physical Therapy and Rehabilitation Education and Research Hospital, Istanbul 34186, Turkey; bsezer91@hotmail.com

**Keywords:** superior cluneal nerve, musculoskeletal ultrasound, chronic low back pain

## Abstract

Superior cluneal nerve (SCN) entrapment is an underrecognized cause of chronic low back pain, and musculoskeletal ultrasound has recently gained attention as a potential diagnostic tool. In a recent pilot study, an indirect ultrasonographic triad consisting of thoracolumbar fascia thickening, iliac crest enthesophytes, and Copeman nodules was proposed as a potential diagnostic indicator of SCN entrapment. In this comment, we discuss several anatomical and methodological considerations regarding the ultrasonographic evaluation of SCN entrapment. Based on previous anatomical studies and our own ultrasonographic experience, we emphasize that the principal role of ultrasonography in SCN entrapment is to facilitate accurate ultrasound-guided diagnostic nerve blocks by identifying the target anatomy. Although direct visualization of the SCN may provide supportive anatomical information, the diagnosis should remain based on the clinical presentation and, when indicated, confirmation by an ultrasound-guided diagnostic nerve block. Further multicenter prospective studies are needed to establish standardized ultrasonographic diagnostic criteria for SCN entrapment.

The superior cluneal nerves (SCNs) are pure sensory nerves originating from the lateral branches of the dorsal rami of T11-L5 nerve roots. They pass between the thoracolumbar fascia and the posterior iliac crest (PIC) and terminate over the gluteal fascia. They provide sensory innervation to the lower lumbar and gluteal region. There are at least three branches of SCN that pass over the PIC, and these are the medial, intermediate, and lateral branches. Anatomical studies have revealed that the medial branch of the SCN (mSCN) passes 6–7 cm away from the midline on the PIC [1,2,3]. Some branches of the SCN, usually its medial branch, pass through an osteofibrous tunnel in the space surrounded by the iliac crest and thoracolumbar fascia. This narrow tunnel is a potential site for SCN entrapment. Since the mSCN crosses the iliac crest towards the midline, where the thoracolumbar fascia is thicker, it is known to become entrapped more frequently than the other branches, making it clinically more relevant [4]. Because of its small size and anatomical variability, the anatomy of the SCN remains incompletely understood.

The diagnosis is based on clinical findings. Helm et al. [5] recommended the use of a “clinical triad” in the diagnosis of SCN entrapment. This clinical triad includes deeply aching and poorly localized low back pain with a variable pattern of referred pain in the buttocks and/or legs, the presence of a tender point at the PIC or caudal to the posterior superior iliac spine, and relief of symptoms by injection of low-dose local anesthetics at the tender point [5].

This clinical triad remains highly practical and useful in daily clinical practice, particularly in cases where direct ultrasonographic visualization of the SCN cannot be achieved. Nevertheless, with high-resolution ultrasound, appropriate patient selection, proper scanning technique, and sufficient operator experience, direct visualization of the SCN is indeed possible [6]. Therefore, when direct visualization is feasible, the diagnostic role of indirect ultrasonographic findings should be interpreted with caution.

Although Iudicelli et al. [7] proposed an indirect ultrasonographic triad due to the small caliber and anatomical variability of the SCN, these findings should be considered supportive rather than primary diagnostic markers. When direct visualization of the nerve is achievable, relying predominantly on indirect findings such as thoracolumbar fascia thickness, iliac crest enthesophytes or Copeman nodules may not be necessary for the diagnosis of SCN entrapment. Furthermore, these indirect ultrasonographic findings may not be specific to SCN entrapment and can also be encountered in other chronic low back pain conditions. Moreover, because the study compared patients with SCN entrapment only with healthy controls rather than with patients with chronic low back pain due to other causes, the specificity of these findings for SCN entrapment cannot be established. In addition, the absence of confirmation by an ultrasound-guided diagnostic nerve block represents another important limitation when interpreting the reported findings.

In our previous study, we visualized the SCN using a standardized ultrasonographic protocol with either a 7–12 MHz linear transducer or a 1–5 MHz multifrequency curvilinear (convex) transducer (Logiq P5, GE Healthcare, Little Chalfont, Buckinghamshire, UK) [6]. Patients were examined in the prone position, and the transducer was placed over the PIC at the point of maximal tenderness and a positive Tinel’s sign, typically located 6–7 cm lateral to the midline. In patients with a low body mass index, a high-frequency linear transducer is generally sufficient for direct visualization of the SCN. However, in our previous study, a low-frequency curvilinear transducer was preferred in some patients to facilitate better visualization of the PIC, a key anatomical landmark, particularly in the presence of increased soft tissue thickness. The SCN was identified as a hypoechoic mono- or oligo-fascicular structure with a typical fascicular appearance, coursing over the PIC in close relationship with the thoracolumbar fascia and within the erector spinae musculature. Identification was based on its characteristic anatomical course, its relationship to the PIC and thoracolumbar fascia, and dynamic real-time scanning. Adjacent vascular structures were excluded using color Doppler imaging to minimize the possibility of misidentification (Figure 1).

Although direct visualization of the SCN represents an important advance, visualization alone does not establish the diagnosis of SCN entrapment. The diagnosis should primarily rely on a compatible clinical presentation and physical examination, with confirmation by an ultrasound-guided diagnostic nerve block when clinically indicated. In current clinical practice, ultrasonography mainly serves to identify the target anatomy and guide diagnostic and therapeutic nerve blocks. Therefore, ultrasonographic findings should always be interpreted in conjunction with the clinical evaluation and the patient’s response to an ultrasound-guided diagnostic nerve block [8].

Furthermore, the diagnostic value of Copeman nodules also deserves consideration. Copeman nodules are fibro-adipose subcutaneous nodular formations traditionally described as herniations of fat tissue through the superficial fascia. However, recent anatomical evidence suggests that these lesions are more likely fibro-adipose nodules or lipomatous lesions located beneath the superficial fascia, without clear evidence of true fascial herniation, thereby challenging the traditional definition of Copeman nodules [9]. Although these nodules have been associated with chronic low back pain, many remain asymptomatic or non-tender. Therefore, their presence alone may not be sufficiently specific for the diagnosis of SCN entrapment, and the exact nature of ultrasonographically detected nodular lesions remains uncertain.

Beyond the structural findings, the underlying pain mechanism also deserves consideration. The thoracolumbar fascia is richly innervated by Aδ and C nociceptive fibers, and structural alterations such as thickening and reduced elasticity may contribute to peripheral sensitization and nociceptive low back pain [10]. However, this mechanism alone may not fully explain the well-localized pain observed in the SCN region or the characteristic clinical features of nerve entrapment syndromes. Therefore, distinguishing fascia-related nociceptive pain from neuropathic pain secondary to SCN entrapment remains clinically important, as these conditions may require different diagnostic and therapeutic approaches.

In our previous study, pain related to SCN entrapment radiated to the leg in approximately 75% of patients, whereas neuropathic symptoms were detected in only 29.2% according to neuropathic pain assessment tools [6]. These findings suggest that even in the presence of nerve entrapment, clinical manifestations may be heterogeneous and not exclusively neuropathic in nature. Further studies are needed to better clarify the relationship between fascial abnormalities, SCN entrapment, and pain phenotypes.

In conclusion, as the role of fascial abnormalities, Copeman nodules, and direct nerve visualization in SCN entrapment remains controversial, further multicenter prospective studies with larger cohorts, standardized ultrasonographic protocols, clearly defined diagnostic thresholds, and longitudinal follow-up are needed to validate these findings, establish standardized ultrasound criteria, and improve diagnostic and therapeutic approaches for this underrecognized cause of chronic low back pain.

## Figures and Tables

**Figure 1:**
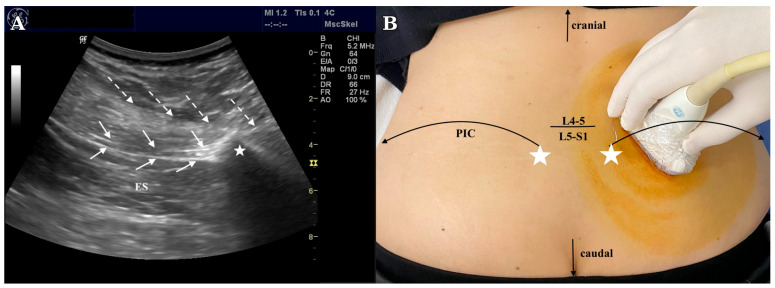
(**A**) Longitudinal ultrasonographic image demonstrating direct visualization of the SCN. The SCN (solid arrows) appears as a hypoechoic monofascicular structure coursing within the erector spinae muscle (ES) before passing between the thoracolumbar fascia (TLF; dashed arrows) and the posterior iliac crest (PIC; white star). Ultrasonographic visualization of the SCN. Adapted from Kiral et al. [6], with permission. (**B**) Patient positioning and ultrasound probe placement for longitudinal ultrasonographic examination of the SCN. The posterior superior iliac spine (PSIS; white stars), the PIC, and the L4–L5/L5–S1 levels are shown as surface anatomical landmarks to facilitate localization of the SCN. Cranial and caudal orientation are indicated.

## Data Availability

No new data were created or analyzed in this study. Data sharing is not applicable to this article.
